# Food anaphylaxis in the United Kingdom: analysis of national data, 1998-2018

**DOI:** 10.1136/bmj.n733

**Published:** 2021-03-19

In this paper by Baseggio Conrado and colleagues (*BMJ* 2021;372:n251, doi:10.1136/bmj.n251, published 17 February 2021), the words “confirmed” and “suspected” should be transposed in two places: (1) the seventh sentence in the results of the abstract, and (2) the fourth sentence under the heading “Fatal food induced anaphylaxis” in the main text.

